# Utilization of transitional care management services and 30-day readmission

**DOI:** 10.1371/journal.pone.0316892

**Published:** 2025-01-03

**Authors:** Eun Ji Kim, Kevin Coppa, Sara Abrahams, Amresh D. Hanchate, Sumit Mohan, Martin Lesser, Jamie S. Hirsch

**Affiliations:** 1 Department of Medicine, Donald and Barbara Zucker School of Medicine at Hofstra/Northwell, Hempstead, NY, United States of America; 2 Institute of Health System Science, Feinstein Institutes for Medical Research, Manhasset, NY, United States of America; 3 Department of Information Services, Data Science and Predictive Analytics, Northwell Health, New Hyde Park, NY, United States of America; 4 Department of Medicine, University of California, San Francisco, San Francisco, CA, United States of America; 5 Department of Social Sciences and Health Policy, Wake Forest School of Medicine, Winston-Salem, NC, United States of America; 6 Department of Medicine, Vagelos College of Physicians & Surgeons and Department of Epidemiology, Division of Nephrology, Mailman School of Public Health, Columbia University, New York, NY, United States of America; 7 Department of Biostatistics, Feinstein Institutes for Medical Research, Manhasset, NY, United States of America; Baotou Medical College, MONGOLIA

## Abstract

Transitional care management (TCM) visits have been shown to reduce 30-day readmissions, but it is unclear whether the decrease arises from the TCM visit itself or from clinic-level changes to meet the requirements of the TCM visits. We conducted a cross-sectional analysis using data from Northwell Health to examine the association between the type of post-discharge follow-up visits (TCM visits versus non-TCM visits based on billing) and 30-day readmission. Furthermore, we assessed whether being seen by a provider who frequently utilizes TCM visits or the TCM visit itself was associated with 30-day readmission. We included adult patients hospitalized to Medicine service and subsequent follow-up visits within two weeks of discharge between February 24, 2018, and February 24, 2020. We examined 1) post-discharge follow-up visit type (TCM visit versus non-TCM visit) and 2) provider characteristics (frequent TCM visit utilization or not). The primary outcome was unplanned hospital readmission within 30 days following hospital discharge. After propensity matching, TCM follow-up visits were associated with decreased 30-day readmissions (hazard ratio = 0.74 [0.63–0.88]) compared to non-TCM visits. Among patients with non-TCM follow-up visits, those seen by a provider who frequently used TCM visits had decreased odds (OR = 0.84 [0.71–0.99]) of 30-day readmission compared to those seen by providers who did not use TCM visits regularly. Among patients who followed up with providers who frequently use TCM visits, TCM visits were associated with decreased 30-day readmission compared to patients with non-TCM visits (OR = 0.78 [0.62–0.98]). The study has limitations, including the health system database not capturing all out-of-network follow-up visits. The reduction in 30-day readmission associated with TCM visits likely arises from both the visit itself and being seen by a provider who frequently uses TCM visits.

## Introduction

Hospital readmissions account for one-fifth of all Medicare expenditures, and one in five Medicare beneficiaries are readmitted within 30 days of hospital discharge [1]. To reduce the cost of preventable readmissions, the Centers for Medicare and Medicaid Services (CMS) established the Hospital Readmissions Reduction Program (HRRP), in which hospitals with readmission rates higher than the targeted rate for six medical and surgical conditions incur financial penalties through reductions in their reimbursements. Subsequently, hospitals and healthcare systems have systematically improved their discharge processes and designed efficient post-discharge care by establishing multidisciplinary teams, coordinating discharge processes, improving patient activation, offering medical home visits, and coordinating post-discharge follow-up visits [2–7]. In particular, post-discharge follow-up visits have been associated with decreased readmission rates, cost, and mortality [8–10].

To further improve post-discharge transition of care, CMS has incentivized post-discharge follow-ups through transitional care management (TCM). This program reimburses providers with higher relative value units (RVUs) compared to routine follow-up if they contact patients within 48 hours of hospital discharge and have a follow-up visit within 2 weeks of discharge [11]. The 48-hour patient contact allows for clinical evaluation of a patient’s status to identify any medical concerns and provides an opportunity to intervene and address common post-discharge complications, such as medication discrepancy or acute decompensation [12]. Many studies examining TCM visits have focused on utilization, demonstrating that TCM visits remain underutilized [8,13–15]. Most studies examining the association between post-discharge follow-up visit type (TCM versus non-TCM visits) have shown reductions in 30-day readmission; however, some studies showed no association between TCM visits and 30-day readmissions [10,16–20]. Lastly, many existing studies are limited to either Medicare data or by small sample sizes, and little is known whether there are spillover effects for all the patients in the hospital system [21].

As more hospitals and health systems are interested in reducing preventable readmission through utilization of the TCM visit, it is important to examine whether the utilization of TCM billing codes is associated with 30-day readmission. Furthermore, it is important to examine whether the findings of the TCM follow-up visits arise from the TCM visit itself or from clinical transformations needed to meet the visit requirements. We therefore examined the association between the type of post-discharge follow-up visits within 2 weeks of discharge and 30-day readmission. We identified post-discharge follow-up visits to be TCM visits if the visits were billed as CPT codes 99495 and 99496. We defined non-TCM visits as post-discharge follow-up visits within 2 weeks of discharge without TCM billing codes. We examined if the reduction in 30-day readmission arises from the TCM visit itself or from provider- or clinic-level process changes that have ensured TCM visit components are met.

## Methods

### Data source

Northwell Health is a large health system in New York with 23 hospitals and over 700 ambulatory facilities. Data for this study was obtained from the enterprise inpatient electronic health record (EHR; Sunrise Clinical Manager, Allscripts, Chicago, IL), capturing data from 13 inpatient hospitals, and the enterprise ambulatory EHR (Touchworks, Allscripts, Chicago, IL), which is utilized at over 450 ambulatory locations.

### Study population

We first queried the inpatient EHR database to obtain data on eligible inpatient discharges between February 24, 2018, and February 24, 2020. We only included admissions to the medicine service for patients 21 years or older. We excluded admissions lasting less than 24 hours and patients who died during their index hospitalization. For patients with multiple hospitalizations, we randomly chose one inpatient admission as the index hospitalization in order to avoid the overrepresentation of patients with multiple admission and follow-up pairs.

Data on post-discharge follow-up visits were obtained from the ambulatory EHR database. We identified patients who arrived to the health system’s general medicine and medical sub-specialty follow-up appointments, including acute visits. We only included post-discharge follow-up visits within two weeks of discharge in order to make an appropriate comparison between groups, as TCM billing is dependent on that timeframe. We used the health system Enterprise Master Patient Index to link patients’ inpatient admissions with their ambulatory visits. The ambulatory data included visits within 14 days of the index discharge date. We excluded patients readmitted prior to their scheduled follow-up visits because we could not predict whether they would show up to their visits or whether their visits would be billed as TCM visits or not. Our previous analysis has shown that the number of patients readmitted before their scheduled visits is low (<1%) at our health system [22].

### Outcomes

The primary outcome was unplanned hospital readmission within 30 days of hospital discharge. We identified index admissions and used the date of patients’ subsequent admissions to calculate the number of days to readmissions. We identified planned readmissions using a standard EHR indicator and excluded them from the analysis. We also included a secondary outcome that included patients with unplanned 30-day readmissions or patients who died after they had post-discharge follow-up visits.

### Transitional Care Management (TCM) visits

We identified TCM visits based on billing codes (*Current Procedural Terminology* codes 99495 and 99496). In order to bill post-discharge visits as TCM visits, it is required that patients are contacted within 48 hours and followed up within 2 weeks of hospital discharge [11]. Patients might have had the contact and follow-up visits within 2 weeks, but physicians or advance care providers might not utilize the code. For this study, we are interested in the utilization of TCM billing codes in post-discharge visits within 2 weeks of hospital discharge. We excluded patients who had TCM visits to non-medicine specialties (i.e., surgery, neurology). We included any post-discharge subsequent visits (including return patient appointments, new patient appointments, acute care appointments, follow-up appointments, or hospital follow-up appointment) that are not billed with CPT codes 99495 and 99496 as non-TCM visits. For patients with multiple follow-up appointments within 2 weeks of discharge, we used the earliest appointment post-discharge as the visit of comparison. Therefore, if a patient’s earliest appointment was non-TCM followed by TCM, we identified the patient as non-TCM.

### Covariates

We included demographic and clinical features associated with 30-day readmissions, including patient age, sex, race/ethnicity, English proficiency, LACE Index score, discharge disposition, primary discharge diagnosis, provider type (general medicine versus medicine specialist), and time period to the follow-up visit [9,23–25]. Race and ethnicity was categorized as non-Hispanic White, non-Hispanic Black, Hispanic, Asian, Other/multiracial, and Unknown. The LACE index (which includes Charlson Comorbidity Index) has been used to predict the risk of unplanned readmission within 30 days after hospital discharge in both medical and surgical patients [26]. We only included patients discharged to home.

We also categorized providers based on whether they are TCM utilizers or not: providers who billed TCM visits eight or more times (top 1% in TCM utilization) during the study period were categorized to be TCM providers. Otherwise, providers were identified to be non-TCM visit utilizers.

### Statistical analysis

Patient characteristics by post-discharge follow-up visit type (TCM versus non-TCM visits) are reported as a number with percentage or mean with standard deviation, where appropriate. We used the chi-squared test for categorical variables and t-test for continuous variables across all groups to test for differences by visit type. Next, we used 1:1 nearest neighbors propensity-score matching method, using the patients in the TCM group as a reference [27]. The model included baseline demographic and clinical information–age, sex, race/ethnicity, English proficiency, LACE Index score, discharge disposition, primary discharge diagnosis, and timing of appointment after discharge. We assessed the covariate balance after propensity score matching by examining the standardized mean difference. We assessed PSM overlap and found that the propensity score distributions were closely identical.

We compared 30-day readmissions between patients with TCM visits and propensity-matched non-TCM individuals using Pearson chi-squared test. We ran a Cox proportional hazard model to examine the risk of 30-day readmission by post-discharge follow-up visit type (TCM versus non-TCM visits) [25]. We used Cox proportional hazards regression to compare 30-day readmission risk by patient’s follow-up visit types [28]. Next, we performed Cox proportional hazards regression among patients with non-TCM visits to identify characteristics associated with 30-day readmission. We were particularly interested in whether there was an association between provider utilization of TCM visits and 30-day readmissions. Then, we ran Cox proportional hazards regression among patients who had visits with providers or clinics who frequently use TCM visits to examine whether TCM visit was associated with 30-day readmission. Lastly, we conducted an inverse probability of treatment weighting and used the resulting data frame into a matched logistic regression. All analyses were performed using the R programming language, version 3.5.0 (R Foundation for Statistical Computing, Vienna, Austria). A p-value < 0.05 was considered significant. The data were accessed September 4, 2020 for this study. The data contained information that could identify individual participants during or after data collection. Due to the retrospective nature of the data, consent was not obtained. This study was approved by the Feinstein Institutes for Medical Research at Northwell Health’s Institutional Review Board.

## Results

We identified a total of 11,391 patients who were discharged and had an outpatient appointment within two weeks of discharge. Following propensity matching, there were 1,881 patients in each group (TCM and non-TCM). The observed 30-day readmission for patients with TCM follow-up visits was 8.4% and the non-TCM follow-up visit was 13.9% (p-value<0.001) (Table 1). Being seen via the TCM pathway was associated with decreased risk of 30-day readmission (hazard ratio (HR) = 0.46 [95% CI 0.39–0.55]) (Table 2).

**Table 1:** Patient characteristics, before and after propensity score matching*.

		Before PSM		After PSM			
	All appointment	Non-TCM	TCM	Non-TCM	TCM	P-value	SMD
	(n = 11,391)	(n = 9502)	(n = 1889)	(n = 1881)	(n = 1881)		
30-Day readmission	1395 (12.25)	1237 (13.0)	158 (8.4)	262 (13.9)	158 (8.4)	<0.001	
Age, mean (SD)	66.4 (16.5)	65.9 (16.5)	69.0 (16.2)	69.1 (15.0)	68.9 (16.2)	0.76	0.01
Race (%)						0.60	0.054
White	7026 (61.7)	5705 (60.0)	1321 (69.9)	1316 (70.0)	1313 (69.8)		
Black	1775 (15.6)	1546 (16.3)	229 (12.1)	246 (13.1)	229 (12.2)		
Asian	745 (6.5)	683 (7.2)	62 (3.3)	47 (2.5)	62 (3.3)		
Other	1547 (13.6)	1323 (13.9)	224 (11.9)	222 (11.8)	224 (11.9)		
Unknown	298 (2.6)	245 (2.6)	53 (2.8)	50 (2.7)	53 (2.8)		
Female	5764 (50.6)	4706 (49.5)	1058 (56.0)	1017 (54.1)	1050 (55.8)	0.29	0.035
English proficient	10390 (91.2)	8632 (90.8)	1758 (93.1)	1757 (93.4)	1750 (93.0)	0.70	0.015
LACE Index score	11.00 [8.00, 14.00]	11 [8, 14]	11 [8, 13]	11.00 [8.00, 13.00]	11.00 [8.00, 13.00]	0.59	0.026
CCI score	6.00 [4.00, 8.00]	6 [4, 8]	6 [4, 8]	6.00 [4.00, 9.00]	6.00 [4.00, 8.00]	0.05	0.087
Appointment time (# of days since discharge)	7.16 [4.28, 10.31]	7.28 [4.34, 10.42]	5.43 [3.31, 8.35]	5.38 [3.27, 8.36]	5.45 [3.32, 8.35]	0.39	0.022
# of days from appointment to readmission	8.94 [4.01, 14.62]	8.89 [4.06, 14.52]	9.50 [3.82, 16.94]	9.57 [3.93, 15.62]	9.50 [3.82, 16.94]	0.90	0.007
# of days from discharge to readmission	15.83 [10.73, 21.57]	15.83 [10.78, 21.52]	15.91 [9.96, 21.93]	15.14 [9.24, 20.77]	15.91 [9.96, 21.93]	0.48	0.067

*n (%) for categorical variable and median [q1 and q3] for continuous variable.

**Table 2:** 30-day readmission risks among patients with post-discharge follow up within 2 weeks of discharge* (HR = Hazard Ratio).

	Propensity score matched		Non-TCM Appointments		Inverse probability of treatment weighting	
	(n = 3,762)		(n = 9,502)		(n = 11,391)	
	HR [95% CI]	P-Value	HR [95% CI]	P-Value	HR [95% CI]	P-Value
TCM	0.46 [0.39–0.55]	< 0.001			0.60 [0.49–0.73]	<0.001
Provider: TCM utilizer	1.01 [0.93–1.11]	0.77	1.05 [0.94–1.17]	0.42		
Clinic: TCM utilizer	0.88 [0.75–1.04]	0.14	0.76 [0.64–0.92]	0.004		

*Adjusting for age, sex, race, English proficiency, LACE index, Charles Comorbidity Index, and admitted hospitals. TCM utilizer is defined as billing at least one visit as a TCM visit during the study period.

Among patients with non-TCM visits (n = 9502), being seen at clinics that used TCM visits was associated with decreased risk of 30-day readmissions (HR = 0.76 [0.64–0.92], p-values = 0.004) compared to non-TCM utilizing clinics. However, non-TCM visits conducted by providers who used TCM visits were not associated with decreased risk compared to non-TCM utilizing providers (HR = 1.05 [0.94–1.17], p = 0.42). There were 37 patients who had post-discharge follow-up visits and died within 30 days of discharge. 7 Patients had TCM visits (7/1889 = 0.37%) and 30 had non-TCM visits (30/9502 = 0.32%). For the secondary outcome, which included patients readmitted within 30 days or who died after having post-discharge follow-up visits, the study findings did not change. We found a similar reduction in readmission risk for the TCM patients (OR = 0.60 [0.49–0.73], p<0.001) with inverse probability of treatment weighting (n = 11,391).

## Discussion

We found that TCM follow-up visits were associated with a lower 30-day readmission risk compared to non-TCM follow-up visits in the same 2-week post-discharge time period. Among patients with non-TCM post-discharge visits, being seen at clinics with TCM utilization was associated with reduced 30-day readmission. Practices that utilize TCM visits probably have designated clinical staff who are accustomed to coordinating post-discharge visits. It is possible that these practices may be more conscious about 30-day readmissions and may spend more time and effort in addressing post-discharge complications. The result also showed that allocating infrastructure and clinical staff dedicated to ensuring safe post-discharge care can also benefit even patients with non-TCM follow-up visits.

The main difference between the TCM visits and non-TCM visits was whether there was documentation of contact by clinical staff within 48 hours of discharge. This suggests that having clinical staff contact patients within 48 hours of discharge is crucial in reducing 30-day readmission, probably through early identification of patients with post-discharge complications. During the clinical contact, staff reviews and assesses the patient’s hospital course, post-discharge medications, and complications. This provides a valuable opportunity to address patients’ complaints or concerns, such as medication adherence (such as not covered by insurance, unclear medication regimen/dosage) and, if needed, escalate the care. Early clinical contact likely enabled patients with urgent medical needs or complex medical conditions to have earlier follow-up benefits. The combination of early contact via telephone followed by a timely follow-up visit also likely allows providers to appropriately manage patients in the outpatient setting rather than patients revisiting emergency departments when they have concerns in the post-discharge period [29,30].

The study finding was similar to other studies examining 30-day readmissions between TCM and non-TCM visits [31]. For this study, we only compared patients who had follow-up visits within 2 weeks of discharge and examined their 30-day readmissions. We excluded patients who were readmitted prior to their post-discharge follow-up visits for multiple reasons. First, not all patients had scheduled follow-up visits (our previous study showed that 36.8% of patients did not have follow-up visits) [22]. Secondly, even if patients had scheduled visits, they might or might not show up to their follow-up visits. Lastly, even if they were to have a visit, we could not predict whether the visit would be a TCM visit. Regarding changes after the COVID-19 pandemic, specifically increased utilization of telehealth post-discharge visits [32,33], it remains unclear whether telehealth visits have similar benefits as in-person visits. In one study, telehealth visit was associated with higher ER visits and hospitalizations compared to in-person visit [34]. However, another study showed that telephone visits had similar 30-day readmissions as post-hospital clinic visits for medium to low-risk patients [35]. The use of telehealth visits will only increase. Consequently, it will be important to ensure that the telehealth visits are appropriately used to ensure that the clinical outcomes are comparable to in-person visits.

Our study has several limitations. Due to the presence of multiple health systems nearby, patients could have had post-discharge follow-up visits at another health system with TCM billing codes. However, a previous study using the health system data showed that the majority of patients hospitalized at Northwell Health hospitals will return to hospitals within the same health system [36]. There also might be a subset of patients belonging to the non-TCM visit group who were contacted by clinical staff within 48 hours of discharge but did not have documentation of the patient contact. This probably reduced the significance of TCM visits in reducing 30-day readmissions. Although no significant differences existed between the two comparison groups after propensity score matching, patients with the non-TCM follow-up visits had later visits compared to patients with TCM visits before the propensity score matching. During the early contact by clinical staff, patients identified to have post-discharge complications were probably asked to make earlier post-discharge follow-up visits. Lastly, there were patients who were admitted before their scheduled appointments. We have excluded these patients from our analysis because we could not predict whether their visits would have been TCM visits or non-TCM visits.

We found that the patients with TCM follow-up visits had significantly lower 30-day readmissions compared to patients with non-TCM follow-up visits. More importantly, being seen at clinics that frequently utilized TCM visits was associated with reduced 30-day readmission among patients with non-TCM visits. As more clinics and health systems implement and adopt TCM follow-up visits in reducing readmission, this study confirms the benefit of the clinical transformation to implement TCM visits. For future studies, it will be important to examine whether TCM visits can address disparities in 30-day readmission to further decrease the risk of 30-day readmission.
